# Towards equity and sustainability of rural and remote health services access: supporting social capital and integrated organisational and professional development

**DOI:** 10.1186/s12913-016-1359-9

**Published:** 2016-04-02

**Authors:** Adrian Schoo, Sharon Lawn, Dean Carson

**Affiliations:** Flinders Rural Clinical School, Flinders University, Mount Gambier, South Australia Australia; Flinders Human Behaviour & Health Research Unit, Flinders University, Adelaide, South Australia Australia; Northern Institute, Charles Darwin University, Darwin, Northern Territory Australia

**Keywords:** Rural workforce, Recruitment and retention, Professional development, Organisational development, Community engagement, Social capital, Rural health service access, Policy development, Chronic conditions, Complex care

## Abstract

**Background:**

Access to rural health services is compromised in many countries including Australia due to workforce shortages. The issues that consequently impact on equity of access and sustainability of rural and remote health services are complex.

**Discussion:**

The purpose of this paper is to describe a number of approaches from the literature that could form the basis of a more integrated approach to health workforce and rural health service enhancement that can be supported by policy. A case study is used to demonstrate how such an approach could work.

**Summary:**

Disjointed health services are common in rural areas due to the ‘tyranny of distance.’ Recruitment and retention of health professionals in rural areas and access to and sustainability of rural health services is therefore compromised. Strategies to address these issues tend to have a narrow focus. An integrated approach is needed to enhance rural workforce and health services; one that develops, acknowledges and accounts for social capital and social relations within the rural community.

## Background

Rural communities generally do not have the same access to health services as their urban counterparts not only as a result of physical distance, but also because of maldistribution of the health workforce [1–3]. Factors that impact on rural health services include challenges to recruitment and retention of health professionals which impact on availability of health services and continuity of care experience and its provision [4, 5]. These, inturn, can threaten the viability of disjointed services, as more rural residents may stop using local services and come to rely their own resources of travel longer distances for services located in regional or metropolitan centres; a situation which contributes to greater prevalence of chronic disease for people living in rural communities, especially where prevention and early intervention and effective coordination of comorbidity is delayed or not addressed [6, 7]. In general, policies to address rural service issues have been fragmented, poorly coordinated, and have had mixed results. The aim of this paper is, based on existing evidence, to question whether there could be a holistic or integrated model of rural service delivery [1, 4, 8] that is more effective in achieving equity and sustainability.

Key to an integrated model is recognition that rural health services should be much more than the provision of various public and private services in isolation from each other [9]. Effective and patient-centred health care requires collaborative practice [10, 11]. Furthermore, rural health services have infrastructure and people (health professionals) who form part of the fabric of local communities and their regions. Health professionals are often community leaders (or potential leaders), knowledge brokers (having relationships with government and academia, for example), and health professional students often actively engage in the social life of the rural community. While the reality often is that rural health professionals and students do not stay for long, and this can impact on the community benefit received [12–14], their very presence still can be seen as providing assets and possibilities for economic, social and cultural sustainability [15–18]. A systematic approach to rural and remote health services should therefore have a community wide perspective, and not just be limited to those directly involved in health treatment [19].

### Towards an integrated model – social capital, social relations and organisational development

**Fig. 1:**
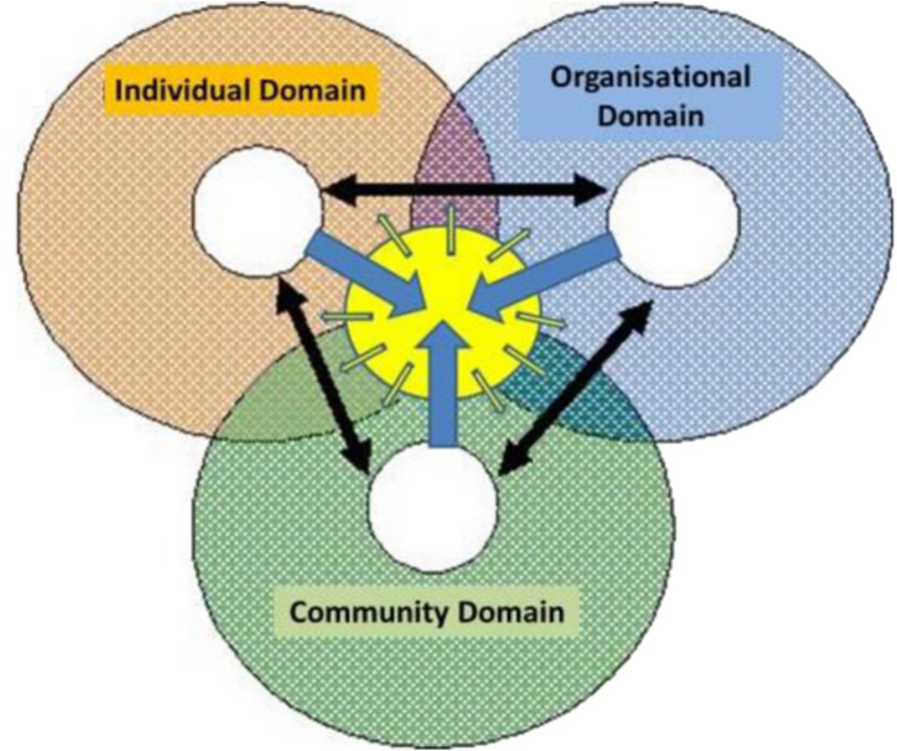
Conceptual health workforce and health service model: Needs, concerns and influence (Adapted from Schoo et al. [20])

Previous work has proposed an interactive conceptual health workforce and health service model (Fig. 1) with three domains (personal or individual, organisational and community) represented as circles of concern and circles of influence [20], and with tension between these in relation what each concerns and what can be influenced. Systems thinking, communication and leadership are enablers that can expand the circle of influence within each domain, and also bring the three together with enhanced stakeholder ownership and associated sustainability of outcomes. Likewise, Worley [18] has proposed a symbiotic clinical education model with the student or studying/advancing health professional at the centre, and four axes (community and government, patients and clinicians, health service and university, and personal principles and professional expectations). These models suggest collaboration between stakeholders for common and community benefit. Although attributes such as social capital and social relations are critical to effective rural health services, theories of social capital and social relationships are largely absent from the health services literature. They are not made explicit as a benefit that could be harnessed; drawing on the nature of rural communities themselves, as solutions.

Social capital includes the norms and networks that bring about collective action for common benefit [21]. Each rural community and its region have a unique blend of individuals that form the actors who live and work there, and network with one another. These actors include health professionals who, for example due to personality or other traits, may be novelty seeking or person or technique orientated [22, 23]. A social capital approach (supported by governance) can build on the uniqueness of each set of networks by facilitating social entrepreneurship. Social capital can be utilised to develop capacity to respond to change in the most effective manner for that community [24]. As such, it is likely to require leadership (including the ability to lead oneself), local and regional engagement, and positive social relations optimise outcomes for rural and remote communities.

Health professionals can contribute to a social capital approach by [25]

- Exploiting their local and extra-local networks to help organise community activities;
- Contributing to social entrepreneurship by helping to negotiate agreements between stakeholders; and
- Providing leadership in identifying opportunities for community development.

There is the potential for health professionals to exercise a mix of social relations within their communities. Health professionals can be engaged through [26]

- **Bureaucratic relations,** which are about hierarchy and asymmetrical control. People are required to meet the personal and collective conditions imposed by formal structures such as in the public and formal education sectors;
- **Market relations,** which can be found in an environment where goods and services are exchanged within a relatively free market place. Market relations allow health professionals to elect the manner in which they wish to operate and network. They provide an environment for service innovation and working between and across the disciplinary borders that are often determined by bureaucratic relations;
- **Associative relations,** which allow people to share common interests and work collaboratively even when not in pursuit of market goals; and
- **Communal relations,** which involve group membership and a common sense of identity. These can be fostered through health professionals’ participation in recreational and social activities.

### Rural and remote health service enhancement

The health care environment is becoming increasingly dynamic and complex in an environment that requires evidence-based practice, value for money and commitment to learning. Learning organisations [27] allow people to continually learn how to work and develop together. Learning organisations engage in systems thinking*,* shared vision, mental models, personal mastery and teamwork/learning [28]. Health professional education needs to include training in these skills. In rural and remote settings, ‘organisations’ could be formed as partnerships between local and regional health professionals and their employers [29] [30]. These could collectively engage in organisational development through an integrated approach to continuing professional development and identification of career pathways as illustrated in the following scenarios.

### Two case scenarios

John is a 58yo man who lives in rural Australia on a small property about 10kms out of town with his wife Christine. The town has a population of approximately 1800 people and is one of a number of small satellite towns to a larger regional centre about 1.5 h’ drive away (approximately 22,000 people) providing core business, health and social care linkages across the region of approximately 60,000 km^2^. The nearest metropolitan centre is approximately 400 km away. John and Christine have no children. About 5 years ago, John was involved in a farming accident and is now wheelchair-bound, relying on his wife for most of his daily personal care needs. Like many people in the area, John and Christine have a well-tended garden, and John gains much pleasure in breeding native and exotic birds. This is a pastime that includes them in regular social get-togethers with other aviculture enthusiasts in the area, including their neighbour Reg, a retired farmer and a member of the local Lions Club who lives 3kms up the road. Members of the local Lions Club have had a number of working bees over the years to help set up the house for John, building a ramp for the house and making the aviaries wheelchair accessible. Tables 1 and 2 illustrate two different regional scenarios.

**Table 1:** Healthcare for John in Region A (fragmented care, with a workforce where sustainability issues are a constant concern and where local residents must rely on only intermittent specialist input, otherwise travel to the city for any acute and specialist care needs

Christine and John visit the GP once a month, usually when they head into town for shopping trips. The practice has served the region for many years; it is a 2-doctor clinic, but that often there is only one doctor because of staff turnover and difficulties in finding locums. Also, one of the GPs will be retiring early next year, leaving only 1 GP, unless they can attract another from outside the region. John receives twice weekly home visits from the local community nursing outreach; they help Christine to shower John on those days, with Christine doing sponge bathes in-between their visits. Otherwise, Christine provides virtually all of John’s hands-on care needs. They are on a waiting list for further support, as packages are limited. Whilst Christine has become somewhat of an expert in warding off skin problems and infections for John, she has found it increasingly tiring to maintain this role and look after the farm. Although she can call on members of the Lions club (and members respond well), and neighbours from time to time, she does not always want to burden them. Also, John had to be admitted several times to the hospital when his condition deteriorated. This is partially due to his lack of physical activity, diet and borderline diabetes condition.
Todd is a physiotherapist who is based in the larger regional centre about 1.5 h’ drive away. He and Ruth the occupational therapist from the region’s community health centre visited John to perform an initial assessment when John came home from hospital. They undertake a review visit separately every 6 months; though they find this increasingly difficult because their resources have been steadily cut over the past 2 years. There was a private physiotherapist but they moved, leaving Todd to cover the whole region. This means that he is on the road a lot and striving to also provide limited services at the community centre. Whilst the community nurses are able to contact John’s GP with any concerns, communication is limited. The primary care nurse at the general practice is part-time and her scope of practice is limited and does not include involvement in chronic condition care planning. Communication between the various health professionals involved in John’s care is limited more broadly, and was reliant on one-way referral to allied health by the GP initially.
Few health professionals in the region have taken on students in the past 5 years. With no links to tertiary education providers and ever growing pressures to see more people over larger distances, they just haven’t had the time. The GPs runs a small practice with few incentives to take on medical students. Their use of chronic disease care planning item numbers is limited. There is some access to telemedicine, although it seems that fewer health professionals are interested to settle in the community or undertake regular visits since this commenced a few years ago.

**Table 2:** Healthcare for John in Region B (progressive in all areas in relation to community engagement, organisational development, workforce retention and continuity/sustainability of health services)

John’s GP coordinated his care from the time of the accident, particularly from the subacute stage. This involves a Team Care Arrangement, enabling the GP to coordinate planned care for John’s various healthcare needs [32]. Christine and John visit the GP once a month, usually when they head into town for shopping trips. The practice has served the region for many years; it is a 2-doctor clinic. Although sometimes doctors are replaced over time, the practice has capacity to receive medical students on placement. John receives fortnightly to monthly home visits from the local community nursing outreach, which is co-located with the community health centre, dependent on his needs. They monitor and check for any signs of pressure sores or infections and work closely with John’s general practice. This involves a 2-way electronic communication alert system for emergencies and stepped-up care, and regular planned fortnightly dialogue between the primary care nurse within the practice and the community nurse to flag any pending issues regarding shared patients. John also receives some help with showering 3 times a week, and daily support for dressing and bed/wheelchair transfers from the local council community care service. The care workers (Janet and Margaret) who deliver this service work out of the local community health centre. Janet was born in the area and did her nurse assistant training through the local TAFE (a dual Certificate IV in Aged Care and Disability). She and her husband have a small property on the other side of town. Margaret moved to the area 6 years ago with her husband and children, who attend the local primary and secondary school, to get away from the pace of city life.
Todd is a private physiotherapist who also does some sessional work at the community health centre and the GP clinic. He works together with Ruth, the occupational therapy case manager at the community health centre, and other health professionals to service the acute and non-acute health needs in the community. This flexibility has enabled both services to react and respond effectively to changing resource pressures for their disciplines. He studied in the city but undertook his final placement in the region and gained a job there once he graduated. He enjoys playing in the local football team. His wife teaches at the local high school. Todd was able to enlist input from his occupational therapy colleague Ruth to provide assessment of John’s showering and transfer needs and home modifications when he first came home after his accident. Ruth provided guidance to the local Lions club to build the ramp and to improve access to the aviaries, with John and Christine’s input about their needs.
Despite their workload Todd and Ruth have been able to provide student placements within their roles. They prefer to have more than one student at the time to allow for the peer learning activities between the students [33]; and, by sharing space and other resources, they succeed in doing that reasonably well. The additional benefits are that this model has a positive impact on service capacity, particularly when students are in their final year, and that it allows Todd and Ruth to do other tasks at times. Another benefit is that some of the final year students over the years have been able to work for a few weeks as a locum after they graduate and before taking up a position elsewhere.
John’s care workers, the occupational therapist, physiotherapist and community nurses, recently undertook training developed and delivered by the aged care provider that operates in the region, in collaboration with its metropolitan office. This involved skills in managing complexity and identifying risk of decline and brought interprofessional staff together across the region to strengthen their networking and communication processes for clients like John.
The TAFE and more recent University link to the region has meant that many of the region’s health and welfare services are better supported to provide student placements across a number of disciplines, and the local residents are assured of a range of good quality health services as they age. Since most healthcare providers in region are relatively small, and have fractional staff appointments or use private service providers like Todd, the providers work together in relation to student placement and health service delivery. Also, the services are part of several managed clinical networks, including a diabetes network that is coordinated by a dietician in the region. The region now has the potential to attract more families seeking a better quality of life and older people coming to retire.
The hands-on support with personal care for John helps Christine significantly so that she can continue much of the work needed to manage the orchard, and John is able to assist her with that by some limited pruning and administrative tasks. The support not only keeps him out of hospital, it’s also an important social contact for John and respite for Christine. The care workers, community nurse and occupational therapist are co-located with the local General Practice and are able to liaise directly with each other, the physiotherapist and the practice nurse of the clinic if they have any particular concerns for John’s health.

## Conclusions

The reality is that communities in regions A and B are likely to be subject to similar pressures such as budget problems, physical distance, youth outmigration, staff turnover and so on. Whilst both need to work around these challenges, the community in region B shows the sort of strong social capital links, as well as strong interprofessional training and networks that have been illustrated in an Australian case study by Munoz and colleagues [31]. As a result, strategies such as sharing physical practice space, utilising professional expertise across sectors and allowing professionals to develop a career in the area of their interest, and community engagement outcomes are likely going to be better and sustained. In contrast, the community in region A still means well, although no thought has been given to how they can use social capital, leadership within the community and entrepreneurship.

The cases raise the question of the extent to which integrated models such as demonstrated in example B can be formally supported by policy that creates the required incentives to optimise processes and outcomes for rural and remote areas - (i) Can policy support collaboration between organisations, communities and providers to provide effective and sustainable rural and remote health services? (ii) If so, can regional health needs and individual needs of health professionals be reconciled with organisational needs? (iii) What are the mechanisms underlying outcomes associated with a multi-faceted approach to social capital, social relations and organisational development (e.g., enhanced health services, health professionals’ perception to make a difference, job satisfaction, not being isolated)?

Disjointed health services are common in rural areas due to the ‘tyranny of distance.’ Recruitment and retention of health professionals in rural areas is problematic and, consequently, access to and sustainability of rural health services is compromised. Strategies to address these issues tend to have a narrow focus and have produced limited positive or lasting results. Policy could support an integrated and evidence-based approach to enhance rural workforce and health services; one that develops, acknowledges and accounts for social capital and social relations within the rural community.
